# Phytoplankton and particle size spectra indicate intense mixotrophic dinoflagellates grazing from summer to winter

**DOI:** 10.1093/plankt/fbac013

**Published:** 2022-03-14

**Authors:** Ovidio García-Oliva, Florian M Hantzsche, Maarten Boersma, Kai W Wirtz

**Affiliations:** Institute of Coastal Systems - Analysis and Modeling, Helmholtz-Zentrum Hereon, Max-Planck-straße 1, Geesthacht 21502, Germany; Institute of Coastal Systems - Analysis and Modeling, Helmholtz-Zentrum Hereon, Max-Planck-straße 1, Geesthacht 21502, Germany; Alfred-Wegener-Institute Helmholtz-Zentrum für Polar- und Meeresforschung, Biologischen Anstalt Helgoland, Helgoland 27483, Germany; Alfred-Wegener-Institute Helmholtz-Zentrum für Polar- und Meeresforschung, Biologischen Anstalt Helgoland, Helgoland 27483, Germany; FB2, University of Bremen, Leobener-Straße, Bremen 28359, Germany; Institute of Coastal Systems - Analysis and Modeling, Helmholtz-Zentrum Hereon, Max-Planck-straße 1, Geesthacht 21502, Germany

**Keywords:** plankton size spectrum, food web, dinoflagellates, mixotrophy, FlowCAM

## Abstract

Mixotrophic dinoflagellates (MTD) are a diverse group of organisms often responsible for the formation of harmful algal blooms. However, the development of dinoflagellate blooms and their effects on the plankton community are still not well explored. Here we relate the species succession of MTD with parallel changes of phytoplankton size spectra during periods of MTD dominance. We used FlowCAM analysis to acquire size spectra in the range 2–200 μm every one or two weeks from July to December 2007 at Helgoland Roads (Southern North Sea). Most size spectra of dinoflagellates were bimodal, whereas for other groups, e.g. diatoms and autotrophic flagellates, the spectra were unimodal, which indicates different resource use strategies of autotrophs and mixotrophs. The biomass lost in the size spectrum correlates with the potential grazing pressure of MTD. Based on size-based analysis of trophic linkages, we suggest that mixotrophy, including detritivory, drives species succession and facilitates the formation of bimodal size spectra. Bimodality in particular indicates niche differentiation through grazing of large MTD on smaller MTD. Phagotrophy of larger MTD may exceed one of the smaller MTD since larger prey was more abundant than smaller prey. Under strong light limitation, a usually overlooked refuge strategy may derive from detritivory. The critical role of trophic links of MTD as a central component of the plankton community may guide future observational and theoretical research.

## INTRODUCTION

Dinoflagellates (Dinoflagellata, Alveolata) are a highly diverse group of organisms (Hackett *et al.,* 2004; Taylor *et al.,* 2008), partially responsible for the formation of harmful algal blooms (Burkholder *et al.,* 2008; Smayda and Reynolds, 2001). The relative success of dinoflagellates as intermittent dominant group in marine and freshwater ecosystems follows from their eco-physiological flexibility (Smayda, 2010a; Hansen, 2011; Gómez, 2012; Jeong *et al.,* 2021): dinoflagellates as a whole can use multiple strategies to occupy diverse ecological niches (Smayda, 2002), in which they obtain, aided by motility, resources such as light, prey or nutrients (Taylor *et al.,* 2008; Smayda, 2010b).

The realized resource strategies of dinoflagellates range from exclusive autotrophy to exclusive heterotrophy, including obligatory or facultative mixotrophy (Schnepf and Elbrächter, 1992; Stoecker, 1999). This trophic diversity places mixotrophic dinoflagellates (MTD) as members of two traditionally recognized functional groups for planktonic protists: (i) phytoplankton and (ii) microzooplankton (Flynn *et al.,* 2013; Mitra *et al.,* 2016). In these regards, MTD can be recognized as part of a more general functional group, the “mixoplankton”—i.e. planktonic protists that can express both phototrophy and phagotrophy (Flynn *et al.,* 2019). As mixoplankton, MTD fulfill multiple roles in the food web, yet the significance of these roles in the dominance of the plankton community is still not well understood (Burkholder *et al.,* 2008; Flynn *et al.,* 2019; Jeong *et al.,* 2021).

MTD are classified based on their main trophic strategy as primarily autotrophs or primarily heterotrophs, depending on the major energetic contribution to growth (Stoecker, 1999; Hansen, 2011; Mitra *et al.,* 2016). Mixotrophy is an adaptive strategy, which reflects the resource availability for autotrophy—i.e. light intensity and inorganic nutrient concentration—as for heterotrophy—i.e. prey availability (Flynn and Mitra, 2009; Mitra and Flynn, 2010; Chakraborty *et al.,* 2017). Heterotrophic resource availability, in turn, partially depends on plankton community structure and biomass, as for example, the plankton size distribution determines the availability of prey for phagotrophic dinoflagellates (Naustvoll, 2000; Wirtz, 2014).

For plankton as a whole, cell size has been considered to act as the master trait that regulates the expression of other traits such as photosynthesis, grazing, nutrient uptake, carbon content etc. (Litchman and Klausmeier, 2008; Finkel *et al.,* 2010; Edwards *et al.,* 2012; Taherzadeh *et al.,* 2017). Moreover, plankton niche formation—i.e. the matching of a class of organism to the use of certain resource/environment—is related to cell size via (non-linear) allometries of respiration, maximal resource uptake rates and mortality due to sinking and grazing (Wirtz, 2013b). The phytoplankton biomass size spectrum is an emergent property of size-dependent processes (Loeuille and Loreau, 2005; Fuchs and Franks, 2010; Taniguchi *et al.,* 2014; Rossberg *et al.,* 2019).

Mixotrophy—as the combination of primary and secondary production in a single organism—alters the flows of energy and nutrients within the planktonic food web (Flynn *et al.,* 2019; Ward and Follows, 2016). These flows are in turn shaped by the size structure of the plankton community (Fuchs and Franks, 2010; Andersen *et al.,* 2015; Chakraborty *et al.,* 2020), where dinoflagellates play diverse roles as predator and/or prey (Jeong *et al.,* 2005a, 2010). These roles are to a large degree determined by cell size (Wirtz, 2012). Although these relations between mixotrophy and cell size in the role of dinoflagellates in the planktonic food web has been already discussed (Jeong *et al.,* 2010; Flynn *et al.,* 2019), the linkage between mixotrophy of dinoflagellates and the plankton size distribution is uncertain.

In aquatic ecosystems, the size distribution settles the structure of the food web (Armstrong, 1999; Zhang *et al.,* 2013), because body size determines the trophic position of an organism (Andersen *et al.,* 2009). The analysis of the plankton size distribution therefore reveals key aspects of predator–prey relationships. First, the size distribution outlines the possible predator–prey pairs based on prey size preference (Fuchs and Franks, 2010; Wirtz, 2012). Secondly, it also specifies both prey and predator abundance (Schartau *et al.,* 2010; Andersen, 2019). In consequence, trophic processes can be effectively assessed in a condensed way by observing the community size spectrum and its changes over time.

Phytoplankton size spectra can be measured through diverse technologies (Lombard *et al.,* 2019). Among these, direct visual identification of microscopic particles is achieved by the imaging in flow cytometer system FlowCAM (Sieracki *et al.,* 1998; Álvarez *et al.,* 2011). This method is fast compared to traditional microscopic enumeration of preserved water samples (v.g. Stoecker *et al.,* 1994; Wiltshire and Manly, 2004; Zarauz and Irigoien, 2008) and enables an assessment of the complete plankton community (Álvarez et al., 2011, 2014; Kydd *et al.,* 2018; Hrycik *et al.,* 2019).

The phytoplankton community in the Southern North Sea is dominated by diatoms and dinoflagellates (Hoppenrath, 2004; Leterme *et al.,* 2006; Baretta-Bekker *et al.,* 2009; Kraberg *et al.,* 2019; Nohe *et al.,* 2020). Among dinoflagellates, MTD play only a secondary role in the plankton dynamics, with a low biomass share compared with strict heterotrophs (Löder *et al.,* 2011). However, MTD may sporadically dominate the water column. Löder *et al.* (2012) reported multi-MTD species blooms—up to 422 μg-C L^−1^ biomass August–September 2007—orders of magnitude larger than the typical biomass concentration of MTD in normal conditions—0.2-30 μg-C L^−1^. These blooms were mainly formed by four ubiquitous direct engulfers MTD taxa (Löder *et al.,* 2012): (i) *Akashiwo sanguinea*, (ii) *Scrippsiella* sp./*Pentapharsodinium*, (iii) *Lepidodinium chlorophorum* (=*Gymnodinium chlorophorum*) and (iv) *Prorocentrum triestinum* (Hoppenrath *et al.,* 2009).

Here, using the FlowCAM analysis, we explore (i) the species succession of dinoflagellates, (ii) changes in size spectra of both MTD and total phytoplankton and (iii) the possible interaction between MTD succession and size spectral changes (i + ii) in the Southern North Sea.

## MATERIAL AND METHODS

### FlowCAM data acquisition

The sampling site is located at the Helgoland Roads station in the Southern North Sea (54° 11.3′N; 7° 54.0′E (Wiltshire *et al.,* 2008)). One liter of water was sampled and prepared for FlowCAM analysis from July until December 2007 in one- or two-week intervals (Hantzsche, 2010).

A portable black and white FlowCAM (www.fluidimaging.com) was used with a green laser beam in the fluorescence triggered image mode (Sieracki *et al.,* 1998) to count only particles that contain chlorophyll. We used the 100 μm flowcell (for size range 2–100 μm) and the 300 μm flowcell (for size range 15–300 μm) in combination with 20$\times$ and 10$\times$ magnification, respectively. To prevent clogging of each used flowcell, the water sample was divided in two sub-samples immediately after sampling: 200 mL was inversely filtered with a 80 μm meshed net funnel for the 100 μm flowcell measurement and 300 mL was inversely filtered with a 250 μm meshed net funnel for the 300 μm flowcell measurement. Particles captured with these flowcell-magnification combinations correspond to a practical size range for particles in the range from 2 to 200 μm (Lombard *et al.,* 2019), covering the range of mixotrophic dinoflagellates and their prey.

The density of fluorescent particles was calculated using the FlowCAM software “Visualspreadsheet” (Version 1.5.16). Particles were visually identified and classified into seven groups: (i) diatoms, (ii) dinoflagellates, (iii) ciliates, (iv) flagellates, (v) coccolithophores, (vi) undefined particles and (vii) detritus. In general, we designate as phytoplankton to the collection of all groups, but detritus. Though some of these groups—e.g. dinoflagellates, ciliates and flagellates—are not *sensu stricto* phytoplankton, we considered them as such given the presence of chlorophyll in their bodies, therefore capable of the use of photosynthesis. We considered all chlorophyll-containing dinoflagellates as mixotrophs, since most chloroplast-bearing dinoflagellates are potential grazers with more or less marked phagotrophic capabilities (Jeong *et al.,* 2010; Löder *et al.,* 2012; Yoo *et al.,* 2009). The same reasoning applies for ciliates and flagellates. In the case of ciliates, our observations could correspond to the mixotrophic species *Mesodinium rubrum* (=*Myrionecta rubra*), the only observed mixotrophic ciliate in Helgoland Roads during all year round (Löder *et al.,* 2010, 2012).

### Size spectra reconstruction and classification

Biovolume and equivalent spherical diameter (ESD, μm) of each particle were calculated using area-based diameter (ABD) measurements. This method closely fits microscope ESD measurements and abundances, even for odd-shaped particles (Álvarez *et al.,* 2014; Kydd *et al.,* 2018; Hrycik *et al.,* 2019). Biovolume was converted to biomass using the scaling formula given by Menden-Deuer and Lessard (2000). We reconstructed biomass size spectra—i.e. the fraction of total biomass per ESD size class—of all phytoplankton and each group following the density estimation method proposed by Schartau *et al.* (2010). The mean ESD and biomass fraction for each group were calculated for each sampling date.

We calculated the fraction relative to the total phytoplankton biomass of the four MTD dominant taxa—i.e. *P. triestinum*, *L. chlorophorum*, *Scrippsiella*/*Pentapharsodinium* sp. and *Akashiwo sanguinea—*by log-normal kernel fitting (Schartau *et al.,* 2010) using the cell size presented by Löder *et al.* (2010, 2012).

We assessed size spectra for each phytoplankton group and date in terms of statistical properties as proposed by Jarque and Bera (1987), Pfister *et al.* (2013) and Gaedke and Klauschies (2017). This method relies on the Skewness–Kurtosis pair $(S,K)$ to classify the shape of size distributions into four common types: (i) normal, (ii) peaked, (iii) skewed or (iv) bimodal. This classification is based on the threshold values outlined by the Jarque–Bera statistics, Pearson’s $S-K$ difference and Sarle’s bimodality coefficient $B$ as described by Gaedke and Klauschies (2017).

The FlowCAM system better captures larger rather than smaller particles (Sieracki *et al.,* 1998). We assessed whether a bias to ameliorate the underestimation of small particles in the size spectra reconstruction. However, we could not find significant effects of the underestimation of small particles or a corresponding bias correction on our results, which are thus shown without bias correction. Further details on the validation and limitations of our FlowCAM methodology are presented in the Supplementary Material (SM2).

### Dinoflagellates feeding kernels and the effect on the phytoplankton size spectrum

Grazing in plankton follows a non-linear function of the predator-and-prey body sizes, in this case expressed by their ESD (Fuchs and Franks, 2010; Banas, 2011; Wirtz, 2012). The optimal prey size—${D}_{\mathrm{opt}}=\log ({\mathrm{ESD}}_{\mathrm{opt}})$—depends on the size of the predating dinoflagellate following a non-allometric scaling (Wirtz, 2012)

(1) \begin{equation*} {D}_{\mathrm{opt}}\left({D}_i\right)={D}_i-0.14, \end{equation*}

where ${D}_i=\log ({\mathrm{ESD}}_i)$ is the logarithmic ESD of the predator dinoflagellate, and the constant value −0.14 is the logarithm of the typical predator-to-prey ratio for dinoflagellates. This value is calculated as the sum of two quantities, one describing all predators, the other specific for a predator group: (i) the logarithm of the scaling factor for a fixed predator-to-prey ratio independent of the predator taxonomy (−1.83) and (ii) the feeding mode of direct engulfers heterotrophic dinoflagellates (1.69) (Wirtz, 2012). The feeding mode is a trait that reflects the activity during feeding and encapsulates all non–size-related terms (Wirtz, 2012). Although dinoflagellates express diverse feeding behaviors—i.e. direct engulfing, pallium or tube feeding (Schnepf and Elbrächter, 1992; Hansen and Calado, 1999; Jeong *et al.,* 2010), here we assumed that all dinoflagellates follow the same feeding mode, thus as direct engulfers (Wirtz, 2012). See also a glossary of terms and equations in Table I.

**Table I:** Glossary of terms and equations used herein

Term	Definition	Symbol	Equation
Equivalent spherical diameter	The diameter of a sphere with equivalent volume than an organism.	$\mathrm{ESD}$	
Predator size	Representative size of a predator as the logarithm of its ESD.	${D}_i$	$\log \Big(\mathrm{ESD}\Big)$
Prey size	Representative size of a prey as the logarithm of its ESD.	${D}_j$	$\log \Big({\mathrm{ESD}}_{\mathrm{prey}}\Big)$
All phytoplankton size spectrum	All present phytoplankton is considered as suitable prey for MTD.	${X}_{\mathrm{all}}\Big({D}_j\Big)$	
MTD size spectrum	All MTD is considered as capable of grazing over all phytoplankton.	${X}_{\mathrm{MTD}}\Big({D}_i\Big)$	
Time	Weeks elapsed from the first sampling date (26 July).	$t$	
Optimal prey size	Logarithm of the optimal prey ESD for a direct engulfer dinoflagellate (Eq. (1)).	${D}_{\mathrm{opt}}\Big({D}_i\Big)$	${D}_i-0.14$
Feeding kernel	Grazing preference of a predator sized ${D}_i$ to a prey sized ${D}_j$ (Eq. (2)).	$f\Big({D}_i,{D}_j\Big)$	${\mathrm{e}}^{-3/2\cdotp{\Big({D}_j-{D}_{\mathrm{opt}}\Big({D}_i\Big)\Big)}^2}$
Grazing flux	Grazing exerted by a predator sized ${D}_i$ to a prey sized ${D}_j$ (Eq. (3)).	$\phi \Big({D}_i,{D}_j\Big)$	${X}_{\mathrm{MTD}}\Big({D}_i\Big)\cdotp f\Big({D}_i,{D}_j\Big)\cdotp{X}_{\mathrm{all}}\Big({D}_j\Big)$
Maximum total grazing flux	Trophic transfer from all phytoplankton to MTD. It is used as normalization constant for the grazing pressure.	${\varPhi}_{\mathrm{max}}$	${\int}_{-\infty}^{\infty }{X}_{\mathrm{all}}^2\Big({D}_j\Big)\cdotp{dD}_j$
Potential grazing pressure	The expected effect of the grazing of MTD over the phytoplankton community (Eq. (4)).	$g\Big({D}_j\Big)$	$\frac{1}{\varPhi_{\mathrm{max}}}{\int}_{-\infty}^{\infty}\phi \Big({D}_i,{D}_j\Big)\cdotp{\mathrm{d}D}_i$
Integral grazing pressure	Degree of overlapping among the phytoplankton distribution and the feeding kernel of the MTD community.	$G$	${\int}_{-\infty}^{\infty }g\Big({D}_j\Big)\cdotp{\mathrm{d}D}_j$
Change in the relative biomass spectra	Change in the biomass spectra observed from $t$ to the following week.	${\varDelta X}_{\mathrm{all}}(t)$	${X}_{\mathrm{all}}\Big(t+1\Big)-{X}_{\mathrm{all}}(t)$
Total fraction of lost phytoplankton	Negative part of the change of the size spectrum difference during the week $t$.	${N}_{\mathrm{lost}}$	${\int}_{-\infty}^{\infty}\min \Big\{{\varDelta X}_{\mathrm{all}}\Big({D}_j\Big)(t),0\Big\}\cdotp{dD}_j$
Feeding-loss index	Change of the biomass size spectra attributed to MTD grazing during the week *t* (Eq. (5)).	$\mathrm{FLI}(t)$	$\frac{-1}{N_{\mathrm{lost}}}{\int}_{-\infty}^{\infty}\min \Big\{{\varDelta X}_{\mathrm{all}}\Big({D}_j\Big)(t),0\Big\}\cdotp g\Big({D}_j\Big)(t)\cdotp{\mathrm{d}D}_j$
MTD feeding kernel	Combined feeding kernel of the MTD community (Eq. (6)).	${f}_{\mathrm{MTD}}\Big({D}_j\Big)$	${\int}_{-\infty}^{\infty }{X}_{\mathrm{MTD}}\Big({D}_i\Big)\cdotp f\Big({D}_i,{D}_j\Big)\cdotp{\mathrm{d}D}_i$

The parentheses immediately after a variable indicate functional dependence. The *i* and *j* subindexes respectively run over the predator and prey size.

Grazing activity slows down when predators feed on sub-optimally sized prey (Wirtz, 2014). This effect is captured by a log-normal feeding kernel $f$, which is centered at the optimal prey size, and symmetrically decreasing in the neighborhoods (Banas, 2011; Wirtz, 2014):

(2) \begin{equation*} f\left({D}_i,{D}_j\right)={\mathrm{e}}^{-3/2\cdotp{\left({D}_j-{D}_{\mathrm{opt}}\left({D}_i\right)\right)}^2}, \end{equation*}

where ${D}_j=\log ({\mathrm{ESD}}_{\mathrm{prey}})$ is the logarithmic prey ESD and 3/2 is the selectivity, which has been derived from simple biomechanical laws and reflects the universal width of the feeding kernel (Wirtz, 2014). Though optimization schemes in prey-size selectivity has been proposed (Tirok *et al.,* 2011), the universal value 3/2 fits well the feeding behavior of dinoflagellates—e.g. the direct engulfer heterotrophic species *Gyrodinium spirale* (Hansen, 1992; Wirtz, 2014).

The feeding kernel $f$ quantifies the strength of the interaction between a predator and its prey. This measure can be extended for entire predator and prey size distributions using the notion of the grazing flux $\phi$ (e.g. Armstrong, 1999; Banas, 2011; Wirtz, 2013a). Here, we assume a simple linear predator–prey interaction, which corresponds to a type I Holling functional response with no saturation. Then, the grazing flux from a prey with size ${D}_j$ as part of the normalized phytoplankton distribution ${X}_{\mathrm{all}}({D}_j)$ toward a predator with size ${D}_i$ with relative contribution given by the MTD distribution ${X}_{\mathrm{MTD}}({D}_i)$ is defined by

(3) \begin{equation*} \phi \left({D}_i,{D}_j\right)={X}_{\mathrm{MTD}}\left({D}_i\right)\cdotp f\left({D}_i,{D}_j\right)\cdotp{X}_{\mathrm{all}}\left({D}_j\right). \end{equation*}

The grazing flux $\phi ({D}_i,{D}_j)$ is a size-based proxy of the grazing activity of a predator, which accounts for both predator and prey community structure. The marginal integrals—i.e. the single integrals over either predator or prey size—of the grazing flux draw meaningful values: (i) the integral over the prey size—–i.e. ${\int}_{-\infty}^{\infty}\phi ({D}_i,{D}_j)\cdotp{\mathrm{d}D}_j$—defines the total grazing from the entire prey spectrum toward a specific predator size and (ii) the integral over the predator size—i.e. ${\int}_{-\infty}^{\infty}\phi ({D}_i,{D}_j)\cdotp{\mathrm{d}D}_i$—defines the grazing pressure of the entire predator spectrum exerted over a specific prey size. The double integral over predator and prey sizes provides the total grazing flux $\varPhi ={\int}_{-\infty}^{\infty}\phi ({D}_i,{D}_j)\cdotp{\mathrm{d}D}_i\cdotp{\mathrm{d}D}_j,$ which describes the total trophic transfer from all prey to the entire spectrum of predators. The total grazing flux reaches its maximum when the prey distribution equals the combined feeding kernels of the predators—i.e. when the prey availability perfectly matches the prey demand. In our case, this is when the feeding kernel of MTD community equals the all phytoplankton distribution ${\int}_{-\infty}^{\infty }{X}_{\mathrm{MTD}}({D}_i)\cdotp f({D}_i,{D}_j)\cdotp{\mathrm{d}D}_i={X}_{\mathrm{all}}({D}_j),$ such that the maximum total grazing flux reads ${\varPhi}_{\mathrm{max}}={\int}_{-\infty}^{\infty }{X}_{\mathrm{all}}^2({D}_j)\cdotp{dD}_j$.With the aid of the grazing flux and its integrals, the effect of the grazing of MTD over the phytoplankton community is calculated by the potential grazing pressure $g({D}_j)$

(4) \begin{equation*} g\left({D}_j\right)=\frac{1}{\varPhi_{\mathrm{max}}}{\int}_{-\infty}^{\infty}\phi \left({D}_i,{D}_j\right)\cdotp{\mathrm{d}D}_i, \end{equation*}

where the denominator is the normalization constant, which was chosen to be the maximum total grazing ${\varPhi}_{\mathrm{max}}$. The integral of the grazing pressure $G={\int}_{-\infty}^{\infty }g({D}_j)\cdotp{\mathrm{d}D}_j$ is bounded between zero and one; in consequence, $G$ can be interpreted as the degree of overlap among the phytoplankton distribution and the feeding kernel of the MTD community. For instance, at $G=0.5$, the feeding kernel of the MTD and the phytoplankton size spectra overlap sharing the 50% of their areas.

The congruence between the biomass lost with the potential grazing pressure is assessed via the feeding-loss index (FLI)

(5) \begin{eqnarray*} \!\!\!\mathrm{FLI}(t)\!\!\!\!\!\!\!\!\!\!\!&&=\frac{-1}{N_{\mathrm{lost}}}{\int}_{-\infty}^{\infty}\!\!\min \left\{{\varDelta X}_{\mathrm{all}}\left({D}_j\right)(t),0\right\}\cdotp g\left({D}_j\right)(t)\cdotp{\mathrm{d}D}_j,\nonumber\\ &&\quad \end{eqnarray*}

where ${\varDelta X}_{\mathrm{all}}(t)={X}_{\mathrm{all}}(t+1)-{X}_{\mathrm{all}}(t)$ is the change in the relative biomass spectra from the week $t$ to the following week $(t+1)$, $g({D}_j)(t)$ is the grazing pressure of the week $t$ and ${N}_{\mathrm{lost}}={\int}_{-\infty}^{\infty}\min \{{\varDelta X}_{\mathrm{all}}({D}_j)(t),0\}\cdotp{\mathrm{d}D}_j$ is the normalization constant, which is the total fraction of biomass of phytoplankton lost during the week $t$. The normalization constant was chosen to interpret the FLI as a measure of the similarity between the distribution of lost biomass and the MTD grazing pressure. The normalization of the potential grazing pressure neglects the relative contribution of MTD in the phytoplankton community; therefore, the MTD percentage within the phytoplankton community is not being considered in the FLI. Thus, in principle, the FLI and MTD biomass fraction are independent variables subject to be compared via a linear regression.

The trophic linkages between the four dominant taxa of MTD—as predators—over other dinoflagellates, other phytoplankton groups and detritus—as prey—were analyzed comparing the feeding kernel of the MTD community ${f}_{\mathrm{MTD}}$ with the time-integrated biomass spectra of each plankton group. The combined feeding kernel of the MTD community is defined by

(6) \begin{equation*} {f}_{\mathrm{MTD}}\left({D}_j\right)={\int}_{-\infty}^{\infty }{X}_{\mathrm{MTD}}\left({D}_i\right)\cdotp f\left({D}_i,{D}_j\right)\cdotp{\mathrm{d}D}_i. \end{equation*}

Finally, prey availability as the percentage of biomass within the feeding kernel was estimated as the integral of the feeding kernel ${f}_{\mathrm{MTD}}$ (Eq. (6)) along the phytoplankton size spectrum. We calculated the prey availability for the four dominant MTD assuming as potential prey (i) all phytoplankton, (ii) all phytoplankton but diatoms, and (iii) detritus.

## RESULTS

**Fig. 1:**
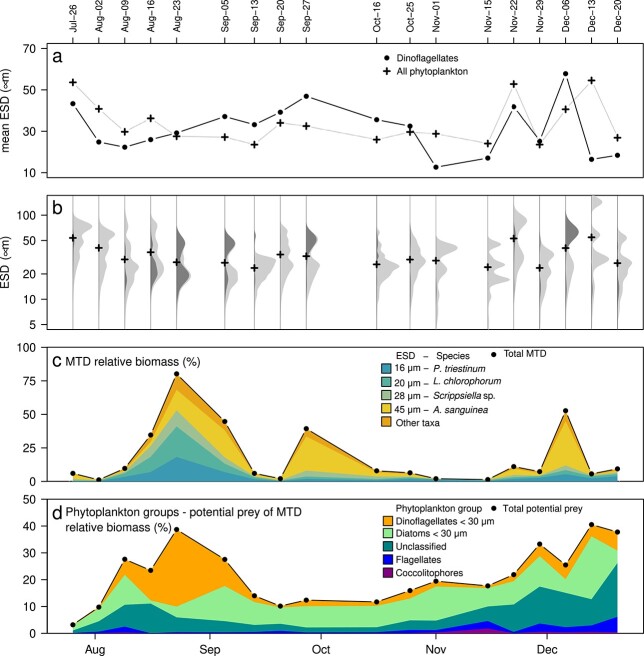
Mixotrophic dinoflagellates (MTD) dynamics from 26 July to 20 December. (**a**) Mean equivalent spherical diameter (ESD) for MTD (dots) and of all phytoplankton (crosses). (**b**) Phytoplankton size spectra (light gray) compared to size spectra of MTD (dark gray). Mean phytoplankton cell size is marked with black crosses. (**c**) Dynamics of total MTD and of the four dominant species. (**d**) Dynamics of the phytoplankton groups potential prey of MTD.

The mean equivalent spherical diameter (ESD) of the phytoplankton community varied between 20 and 60 μm in the period from 26 July to 20 December (Fig. 1a). During the MTD bloom—from 9 August to 27 September—the mean ESD of MTD increased from 20 to 45 μm. From 15 November to 20 December, the size composition of MTD was highly variable.

The recorded size spectra revealed great variability in their functional shape including, e.g. bi- and unimodal size distributions (Fig. 1b). The maxima of the phytoplankton size spectra varied with date and dominant group. When diatoms dominated from 26 July to early 2 August, the maximum of the unimodal biomass distribution was around a cell size of 75 μm. Otherwise, cell sizes at spectral maxima scattered around 25 and 30 μm, especially after MTD dominance (25 μm for 13 September to 20, 20 μm for 16 October and −25, and 30 μm for 1 November). In comparison, the total phytoplankton biomass size spectra were bimodal during MTD dominance, with maxima at 20 and/or 50 μm (Fig. 1b, see Fig. S1 for the normalized MTD size spectra).

Bimodal distributions entailed persistent large dips around 30 μm (Fig. 1b). This minimum size coincides with the (theoretical) optimal prey size of *A. sanguinea* (29 μm) (Table II). Other local minima were located around 15 and 75 μm. For bimodal distributions, the minimum of all phytoplankton size spectrum coincided with the phytoplankton mean size (Fig. 1b, dates 23 August, 5 and 27 September, 22 November and 6 December).

We identified three periods of MTD dominance. First, from 2 August to 13 September, the mean ESD of MTD began smaller and ended larger than the mean phytoplankton ESD (Fig. 1c). The second bloom—from 20 September to 16 October—featured larger MTD than the remaining community. The third period—around 6 December—was also dominated by large MTD with ESD of approx. 60 μm, but at low biomass and chlorophyll concentration (Fig. S2).

The phytoplankton groups considered as potential prey of MTD peaked above 40% during the first and third dominance period (Fig. 1d). Unclassified plankton dominated the first half of August and shifted to small dinoflagellates dominance in 23 August. Between the second and third period of dinoflagellates dominance—i.e. 27 September to 22 November—, small diatoms dominated and then shifted to unclassified plankton dominance in 22 November to the end of our observations.

The biomass of MTD and chlorophyll-a concentration of the entire plankton community (Chl-a) did not follow the same dynamics (Fig. S2). The two aforementioned MTD blooms—maxima on 23 August and 27 September—were only partially visible in the Chl-a signal.

The maxima of the biomass spectrum during MTD dominance coincided with the size of individual dinoflagellate taxa (Table II): *P. triestinum* (17 μm), *Lepidodinium*  *chlorophorum* (20 μm), *Scrippsiella*/*Pentapharsodinium* sp. (28 μm, hereafter only referred as *Scrippsiella* sp.) and *Akashiwo sanguinea* (45 μm). Other MTD taxa made less than the 10% of the MTD biomass in the study period (Fig. 1c).

MTD biomass began to increase on 2 August, reaching 80% of total phytoplankton biomass in 23 August (Fig. 1c). In mid September, a strong decline in MTD biomass insinuated high mortality rates, effects of plankton vertical migration or vertical mixing produced by the tidal cycle (Blauw *et al.,* 2012). Dominance of MTD terminated at 1 November, with less than 1% of total biomass until early December, when the third period of MTD dominance started.

The phytoplankton composition during the first and second MTD dominance period (Fig. 1c) shifted from co-dominance of *P. triestinum*, *L. chlorophorum*, *Scrippsiella* sp. and *A. sanguinea* (August), toward dominance of *A. sanguinea* (September and October). During this period, $\sim 30$ μm ESD diatoms and $\sim 20$ μm unclassified plankton were present. For MTD, the observed species succession was in accordance with the observed change in mean ESD.

**Table II:** Cell size and optimal prey size of dinoflagellates

		Prey ESD (μm)	
Species	ESD (μm)	Theoretical	Observed
*Prorocentrum triestinum*	17	7 (4–14)	1–12
*Lepidodinium chlorophorum*	20	10 (5–20)	5
*Scrippsiella* sp.	28	17 (9–35)	1–12
*Akashiwo sanguinea*	45	29 (15–57)	1–28

Equivalent spherical diameter (ESD) of the four dominant species in this study was reported by Löder *et al.* (2012). Theoretical prey size was calculated using a mechanistic relation (Wirtz, 2012). In parenthesis, feeding kernel widths with preference >0.5 are given (Wirtz, 2014). Ranges in prey size were observed by Jeong *et al.* (2005b), albeit reported with different dinoflagellate ESD (*P. triestinum* 12.6 μm, *S. trochoidea* 22.8 μm and *A. sanguinea* 30.8 μm), and by Ng *et al.* (2017) for *Lepidodinium* sp. (14 μm). The observed prey ESD includes the predation of *Synechococcus* sp. (Jeong *et al.,* 2005a).

Diatoms were overall the most abundant group, with a share of relative biomass between 13% and 94%. From 9 August to 23 August, just before the MTD maximum onset, unclassified phytoplankton (ESD $\sim 20$ μm), small flagellates (ESD $\sim 20$ μm) and coccolithophores (ESD $\sim 4.5$ μm) were present with relative biomass of approx. 10%, 3% and 1.8%, respectively (Fig. S3). Biomass of detrital particles ranged from 10^−2^ to 10^3^ times the biomass of phytoplankton. Mixotrophic ciliates (relative biomass 0.1%) and other groups such as haptophytes, raphidophytes and silicoflagellates were identified, but due to their low biomass share neglected in our analyses (relative biomass <0.01%).

**Fig. 2:**
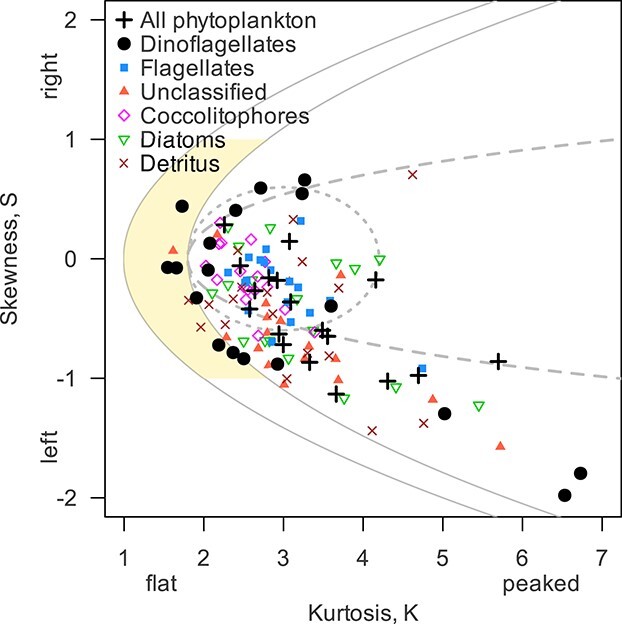
Characterization of biomass size spectra (Skewness–Kurtosis diagram). The lines correspond to the boundaries of three normality indexes: Jarque–Bera test statistics (dotted inner ellipse), Pearson’s *S* − *K* difference (continuous line) and Sarle’s bimodality coefficient (dashed line). The leftmost line is the theoretical limit for the Pearson’s *S* − *K* difference. The shaded area (yellow) corresponds to the bimodality region. Skewness and kurtosis measure the asymmetry and the tail sizes, respectively, using as reference the normal distribution (*S* = 0 and *K* = 3).

The weekly Skewness–Kurtosis diagram (Fig. 2) for all phytoplankton and the seven particle groups size spectra reveals that all phytoplankton size spectra were in general more peaked than a normal distribution (mean kurtosis $K=3.15$) and slightly skewed to the left, thus toward smaller ESD (mean skewness $S=-0.39$). Spectra of dinoflagellates can be categorized as (i) bimodal (7 of 18 observations, mean Sarle’s bimodality coefficient $B=0.89$); (ii) left skewed (4 observations) and (iii) nearly normal distributions (7 observations) (see Fig. S1 for the normalized spectra). Size spectra of other plankton groups were always normal, unimodal, ranging from nearly symmetrical to left skewed distributions (skewness $-1.5<S<0.5$).

**Fig. 3:**
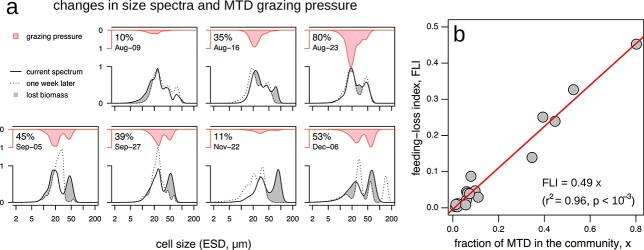
Effects of grazing of MTD over the phytoplankton size spectrum. (**a**) Changes in size spectra and potential grazing pressure of MTD when the fraction of MTD in the community was more than the 10% of the total biomass (value shown in the top left corner). (**b**) Feeding-loss index (FLI) correlated with the fraction of MTD in the community (*x*). The linear regression is significant with a slope of 0.49 ± 0.03 (*n* = 18, *r*^2^ = 0.97, *P* < 10^−3^).

The changes in the size spectra of all phytoplankton are related with the potential grazing pressure of MTD (Fig. 3): the regions in the size spectrum with lost biomass coincide with the regions with greater potential grazing pressure, especially when the fraction of MTD in the plankton community is above the 10% (Fig. 3a). The feeding-loss index—i.e. a metric for the agreement between the MTD grazing pressure and the phytoplankton biomass loss—and the fraction of MTD in the plankton community are significantly correlated (${r}^2=0.97$, $P<{10}^{-3}$) (Fig. 3b). The value of the FLI-to-fraction of MTD slope ($0.49\pm 0.03$) indicates that up to approx. 49% of the biomass lost in a week is potential consequence of grazing of MTD.

**Fig. 4:**
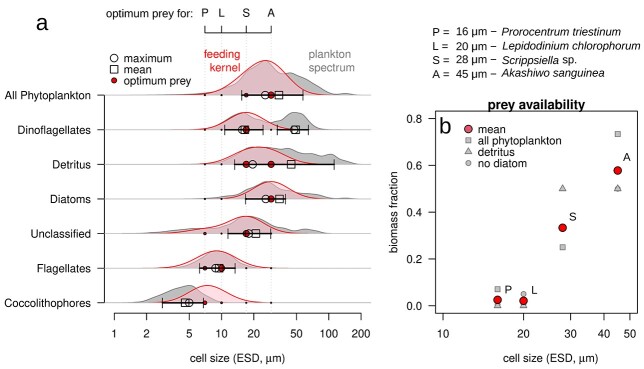
Feeding kernel and prey availability for MTD. (**a**) Comparison of time-integrated biomass size spectra for plankton groups and detritus (gray) with the best fitting MTD feeding kernel (red). Void symbols mark the maximum (circle) and mean (square) size for each group and the error bars the region where the biomass is larger than 50% of the maximum. Solid circles (red) mark the optimum prey size for each dinoflagellate species. The size of the marker is proportional to the overlapping of the feeding kernel with the plankton biomass spectrum. (**b**) Prey availability estimated as the biomass fraction of the time-integrated size spectra within the feeding kernel as function of body size for the four dominant dinoflagellates species. Small symbols (gray) mark different assumptions of potential prey (square = all phytoplankton as potential prey, triangle = only detritus, circle = all phytoplankton but diatoms is potential prey), and big circles (red) show the mean prey availability.

Among all phytoplankton groups, only dinoflagellates exhibit a time-integrated size spectrum that is bimodal, with maxima around 15 and 50 μm (Fig. 4a). The mean size 30 μm coincides with the local minimum of the spectrum. Distributions of coccolithophores and flagellates are approximately normal with maxima at 4.5 and 8.8 μm, respectively. The distribution of diatoms peaked at 30 μm, thus in the gap of the bimodal MTD distribution. Distributions of unclassified plankton and detritus reveal a broad and irregular integrated size distribution.

The comparison of the time-integrated biomass size spectra for each phytoplankton group with the feeding kernel of the MTD (Fig. 4a) suggests the following trophic linkages: (i) *A. sanguinea* feeding over smaller dinoflagellates, detritus, diatoms and unclassified plankton; (ii) *Scrippsiella* sp. feeding over smaller dinoflagellates, detritus, diatoms and unclassified plankton; (iii) *L. chlorophorum* feeding over flagellates and unclassified plankton and (iv) *P. triestinum* feeding over flagellates, unclassified plankton and coccolithophores. In general, prey availability during our study period was higher for large MTD than for small MTD (Fig. 4b): approx. 60% of total biomass (including detritus, diatoms and other phytoplankton taxa) was available as potential prey for *A. sanguinea* and 30% for *Scrippsiella* sp. In comparison, less than 10% of the total biomass was potentially usable by small MTD—*P. triestinum* and *L. chlorophorum*.

## DISCUSSION

The co-occurrence of multiple species and succession of MTD as displayed by our North Sea data has been similarly observed in other coastal seas worldwide. For instance, in Masan Bay (Korea), blooms of *Akashiwo sanguinea* were associated with other species of dinoflagellates, v.g. *Prorocentrum minimum* (Jeong *et al.,* 2005b, 2013). Species succession on Masan Bay also followed a similar pattern as observed in our study: from small non-dinoflagellate plankton toward large dinoflagellates at the end of the blooming phase (Jeong *et al.,* 2013). Also, in Alexandria (Egypt), larger dinoflagellates replaced smaller ones (Labib, 1996; Ismael, 2003). Here, in Alexandria, a clear distinction in trophic strategy and cell size emerged: mixotrophic and autotrophic species were the major biomass contributors (98%) in sizes 30 μm, while heterotrophic species were dominant (90% biomass) in larger cells (50 μm) (Ismael, 2003). Other observations of co-occurring MTD in coastal blooms include Northern California (USA) (White *et al.,* 2014), the Adriatic coast (Bužančić *et al.,* 2016), Chabahar (North of Gulf of Oman) (Koochaknejad *et al.,* 2017) and St. Helena Bay (South Africa) (Ndhlovu *et al.,* 2017).

### A hidden dinoflagellate food web?

In our study, *A. sanguinea* dominance in September was associated with a decrease in biomass of small dinoflagellates. From the comparison of the feeding kernel of *A. sanguinea* (15–60 μm) with the biomass size distribution (Fig. 4), we suggest that this large MTD species could graze on smaller dinoflagellates. Cell size of *Scrippsiella* sp. (28 μm) is close to the theoretical optimal prey size (29 μm). The disappearance of an unclassified plankton group (ESD $\sim 20$μm) during the growth period of *A. sanguinea* may also relate to grazing control. Although direct evidence lacks so far, a significant correlation of FLI with the fraction of MTD in the phytoplankton community (Fig. 3b) in combination with the match of the theoretical feeding kernel with the size spectrum (Fig. 4) is here taken as indirect indication for trophic interaction. Grazing of *A. sanguinea* on small plankton, including other MTD and unclassified plankton, can act as an important driving force of species succession in the Southern North Sea, at least in autumn.

The idea of phagotrophic activity driving MTD blooms and succession—as suggested by Figs 1 and 3—is supported by other studies. *A. sanguinea* (=*Gymnodinium sanguineum*) grazes over small ciliates (ESD $\le$ 20 μm) (Bockstahler and Coats, 1993a, 1993b) and small dinoflagellates, including members of the genera *Prorocentrum* and *Scrippsiella* (ESD 12 and 23 μm, respectively), with a significant impact on the prey populations (Jeong *et al.,* 2005b). For the second bloom and the third dominance period in December, the hypothesis of prevailing phagotrophy in MTD holds if the diet of *A. sanguinea* also includes diatoms and detritus. Both, diatom and detritus size spectra fit into the optimal prey range of *A. sanguinea* (Fig. 4). The effects of dinoflagellate grazing on diatoms have been observed even at large scale (Barton *et al.,* 2013; Sherr and Sherr, 2007).

Detritivory and bacterivory may also support mixotroph blooming by providing an additional carbon source in periods of low light availability (Havskum and Riemann, 1996; Poulsen *et al.,* 2011). Detritivory of copepods and dinoflagellates accelerate detritus recycling (Svensen *et al.,* 2014) and turnover rates of copepod fecal pellets (Kiørboe, 2003). Our observations show that detritus concentrations were high during the study period and increasing in winter, associated with a reduction of water clarity (Fig. 1), a common feature of coastal seas (Andersson and Rudehall, 1993; van Valkenburg *et al.,* 1978). A large fraction of detritus biomass is within the feeding kernel of *A. sanguinea* (Fig. S4), especially during November and December, when detritus mass concentration was 20 times the one of phytoplankton (Fig. 2). As a consequence, detritivory may in part explain the third MTD dominance period (Dec-06, Fig. 1c).

Simulations of ecosystem dynamics of the Southern North Sea show that chlorophyll concentrations are systematically underestimated in winter (Lemmen, 2018; Wirtz, 2019). The underlying model reveals very high skill predicting chlorophyll concentrations in other seasons, but neglects mixotrophy as a “survival” or “refuge” strategy. The possibility of MTD ingesting detrital particles may thus become relevant in current ecological models. The account of “detritivorous phytoplankton” can describe an alternative source of nutrients and carbon for phytoplankton growth in locations and times where strict autotrophy is light limited, i.e. in coastal turbid waters during winter. Under these low light conditions, mixotrophy including detritivory and bacterivory, in combination with other traits such as motility and cyst formation, may provide a growth advantage for MTD over diatoms. Recurrent dominance of MTD over diatoms has frequently been reported (Warns *et al.,* 2013; Spilling *et al.,* 2018), but so far only poorly understood in terms of physiological traits where usually diatoms are more competitive.

Our study also indicates additional predator–prey relations for other MTD. The theoretical prey size ranges of *P. triestinum* (4–14 μm) and *L. chlorophorum* (5–20 μm) include the size spectra of flagellates, unclassified groups and coccolithophores (Fig. 4a). One week before the onset of the first bloom (9 August), the lost biomass—as the potential grazing pressure of MTD— was concentrated around the flagellates mean size, between 5 and 15 μm ESD. In this week, flagellates reach a share of 3% of total biomass, and then this percentage decreased as *P. triestinum* and *L. chlorophorum* grew (16 August), which suggests that these two species grazed on flagellates. In natural ensembles, *P. triestinum* is known to prefer non-dinoflagellate prey (Jeong *et al.,* 2005b), such as small diatoms—*Skeletonema costatum* ESD $\sim 6$ μm (Yoo *et al.,* 2009)— and cyanobacteria—*Synechococcus* sp. ESD $\sim$ 1 μm (Jeong *et al.,* 2005a). The theoretical prey size range of *Scrippsiella* sp. (9–35 μm) includes the unclassified group, detritus and smaller MTD. However, lab grazing experiments report preference toward smaller prey, such as observed for *Scrippsiella trochoidea* in the range 1–12 μm (Jeong *et al.,* 2005a, 2005b) (Table II, Fig. 4).

The grazing of MTD impacts the phytoplankton size spectra by the remotion of prey in specific size classes (Fig. 3). In our study, the biomass of phytoplankton lost in a region of the spectra matches with the potential grazing pressure of the MTD over the phytoplankton community (Fig. 3a). The similarity between these both distributions—measured by the FLI—significantly increases as the fraction of MTD in the community does (Fig. 3b). We take this as evidence of prey remotion by grazing of MTD. In general, MTD might play a major role in the plankton remotion, with considerable clearance rates over algal populations (v.g. Bockstahler and Coats, 1993a; Jeong *et al.,* 2001; Yoo *et al.,* 2010; Kang *et al.,* 2011).

Despite the general good agreement between FLI and the fraction of MTD, inconsistencies arise in some regions of the size spectra. For instance, the biomass lost and the MTD grazing pressure mismatch from 50 to 150 μm ESD in 22 November (Fig. 3a). As this size range is out of the feeding kernel of our MTD, we can refer these artifacts to the grazing of other groups—e.g. fish, copepods, heterotrophic dinoflagellates and ciliates—co-occurring in the ecosystem but not considered in our analyses. The clearance rates of these other groups are rather high compared with the ones of dinoflagellates (Kim and Jeong, 2004; Neuer and Cowles, 1995). Yet dinoflagellates can be more effective in removing prey than, for instance, copepods, when present in high abundances (Kim and Jeong, 2004), condition met in our study during the MTD blooms (Fig. 1). Additionally, the biomass of heterotrophic dinoflagellates and ciliates—whose potential feeding kernel overlap the one of MTD—were low in comparison to the biomass of MTD (Löder *et al.,* 2012).

The trophic interactions, possibly responsible for the observed changes in phytoplankton biomass and size distribution, are based on size preference, which in turn depends on the size of the dominant phagotrophic MTD species. The latter may change due to external factors. For example, the mean size of dinoflagellates in 20 December is larger (60 μm ESD) than the nominal size for *A. sanguinea—*45 μm ESD, after Löder *et al.* (2012). This size shift, previously reported in other *A. sanguinea* blooms, has been linked to high nutrient concentration, mainly phosphorus (Smayda, 2010a), nutrient-induced sexual reproduction (Smalley *et al.,* 2003; Badylak *et al.,* 2017), life cycle (Tang and Gobler, 2015), or with growth rate: during fast-grow periods, dinoflagellates have a smaller and less-variable cell size (Badylak *et al.,* 2014). This size variability is regarded as phenotypic plasticity (Scheiner, 1993) and potentially modifies, among other traits, the optimal prey size of the predator (Berge *et al.,* 2008). Probably, the shift toward larger organisms is the reason why the theoretical prey size and the observed prey range in Table II partially diverge, especially for *A. sanguinea*. It should be noted that the differences between theoretically derived and observed optimal prey ranges also reflect uncertainty in the assumed feeding mode as in the study of Wirtz (2012); the latter is for strict heterotrophic dinoflagellates, while our species are all known as mixotrophic (Hoppenrath *et al.,* 2009; Jeong *et al.,* 2010). These observed differences may guide future studies relating the optimal prey size with feeding traits of dinoflagellates—v.g. feeding mechanism and trophic strategy.

### Trophic strategies of dinoflagellates

Our MTD species may under certain conditions predominantly rely on photosynthesis (Jeong *et al.,* 2005c; Matsubara *et al.,* 2007). For instance, in lab conditions, photosynthesis is the major contributor to total growth rate, while the contributions of grazing are less than 50% for three of our dominant MTD, i.e. *A. sanguinea* 3.6% (Jeong *et al.,* 2021); *S. trochoidea* 35%, calculated after Ng *et al.* (2017); and *P. triestinum* 14.7% (Jeong *et al.,* 2021). However, the relative importance of photosynthesis and grazing in the total growth rate of MTD is highly variable, and some species rely almost completely in photosynthesis—up to 96.4% of the total growth rate—while others do in grazing—up to 100% of the total growth rate (Lee *et al.,* 2014; Jeong *et al.,* 2021).

Despite the low contribution to the total growth rate in lab conditions of our MTD species, mixotrophic grazing is an important factor in the bloom development (Burkholder *et al.,* 2008). For instance, grazing plays a major role during blooms of *A. sanguinea* even if its contribution to the total growth rate remains low: mixotrophic grazing accounts for 30% of prey remotion, with a contribution of up to 11.6% of carbon—3-fold the value reported for lab conditions 3.6% (Jeong *et al.,* 2021)—and 18.5% of nitrogen per cell (Bockstahler and Coats, 1993a, 1993b; Jeong *et al.,* 2005b). Grazing may be especially important in the low light conditions observed during the onset of *A. sanguinea* dominance (2 m of Secchi depth during late September and early October, Fig. S2).

The balanced use of photosynthesis and grazing in a mixotroph should reflect resource availability in terms of light, inorganic nutrients and prey (Stoecker, 1999; Flynn and Mitra, 2009; Hansen, 2011; Andersen *et al.,* 2015; Chakraborty *et al.,* 2017). Resource use might play a fundamental role in the dynamics of plankton communities (Mitra *et al.,* 2014; Stibor *et al.,* 2019; Leles *et al.,* 2021). The importance of grazing in mixotrophs is beyond its contribution to the total growth rate, nutrient—e.g. nitrogen and phosphorus—acquisition being another critical factor that stimulates mixotrophy (Legrand *et al.,* 1998; Johnson, 2015). However, the emergence of a hidden food web over the bloom development facilitates the direct transfer of nutrients and energy within the plankton community.

Size diversity of MTD may regulate the biomass transfer efficiency (García-Comas *et al.,* 2016). This size diversity increases particularly in bimodal distributions. Due to the disentanglement of MTD dynamics from light and nutrients, ambient resource availability may become less critical in driving the species succession during a co-occurring MTD bloom (Jeong *et al.,* 2010), as already proposed earlier (Mitra and Flynn, 2010; Jeong *et al.,* 2013). Mixotrophs in harmful algal blooms might behave primarily as heterotrophs, using photosynthesis only to counter shortages in prey (Flynn and Mitra, 2009). We propose that the size diversity in the MTD community—entailed in our case by a bimodal distribution—allows for an efficient usage of a wide range of prey size groups, from picophytoplankton to diatoms and small MTD.

### Is bimodality a consequence of niche differentiation?

Our observations reveal a great shape diversity of plankton biomass size spectra, in agreement with other studies: in general, multimodal and skewed distributions are more frequent than unimodal and symmetric distributions (Gasol *et al.,* 1997; Havlicek and Carpenter, 2001; Schartau *et al.,* 2010; Álvarez *et al.,* 2011; Gaedke and Klauschies, 2017). At our site, each plankton group occupies a range in the size continuum (Fig. 4a). Except for dinoflagellates, size spectra for whole groups were unimodal, nearly symmetric or slightly left-skewed. Why is the dinoflagellate size spectrum so different?

Theoretical studies suggest high evolutionary stability in bimodal trait distributions (Menden-Deuer and Rowlett, 2014; Peeters and Straile, 2018). Bimodal trait distributions allow for a more sustainable response option compared to a normal distribution, especially if the pressure is imposed around the mean (Perron and Carrier, 1981; Hendry *et al.,* 2009; Weithoff and Beisner, 2019). Bimodality is thought to reflect disruptive selection, i.e. when extreme phenotypes have a fitness advantage over more intermediate phenotypes (Rueffler *et al.,* 2006). For example, in a size-structured community, grazing over the mean size increases the relative fitness on the neighborhoods, sometimes inducing the formation of bimodal size spectra (Hahn and Höfle, 1999; Wirtz, 2013a; Coutinho *et al.,* 2016; Taherzadeh *et al.,* 2019).

Here we propose a link between the dinoflagellate bimodal size distribution and niche differentiation, which in turn results from the large trait diversity of dinoflagellates (Hackett *et al.,* 2004; Taylor *et al.,* 2008; Smayda, 2010a; Gómez, 2012). It is unclear which trait is responsible of this diversification, but the proposed dinoflagellate hidden food web points to trophic interaction. In this scheme, two mechanisms could act simultaneously: (i) the balanced use of photosynthesis and grazing creates two optimal cell sizes for dinoflagellates, one size characteristic for a specialist in photosynthesis and for the other one in phagotrophy, and (ii) the grazing pressure of large on small MTD creates disruptive selection that breaks an originally unimodal into a bimodal distribution. This last mechanism is evidenced in the maximum grazing pressure of MTD (Fig. 3)—approx. 30 μm ESD—which is located in the same region as the dip in the dinoflagellates bimodal distribution (Fig. 4). Trophic strategy might act as the “second master trait”—the first being cell size—for dinoflagellates. Trophic strategy as a secondary master trait could regulate other functional or behavioral traits as motility, migration or morphology (Ault, 2000; Schuech and Menden-Deuer, 2014). The differentiation of trophic niches has been suggested for other mixotrophic groups such as chrysophytes (Lie *et al.,* 2018; Wilken *et al.,* 2020), ciliates (Modenutti *et al.,* 2008), mixoplankton in N-based models (Anschütz and Flynn, 2020) and mixoplankton in temperate seas (Leles *et al.,* 2021). For dinoflagellates, the high inter- and intra-specific diversity and adaptive capabilities make niche differentiation a viable explanation for their frequent bimodal size spectrum (Menden-Deuer and Montalbano, 2015; Luo *et al.,* 2017; Brandenburg *et al.,* 2018; Meng *et al.,* 2019).

As already discussed, size bimodality suggests the existence of two distinct groups, one of more phototrophic and the other of more phagotrophic dinoflagellates. In our analysis, we observed higher prey availability for larger MTD (Fig. 4b) and higher chlorophyll fluorescence for smaller MTD (Fig. S6) during the study period. These trends were paralleled by a change in plankton community structure. Also, light availability was much reduced during large MTD dominance (Fig. S2). These observations together with our theoretical reconstruction of trophic links within a size structured food web indicate that large dinoflagellates might rely more in grazing than smaller species. The relationship between size and trophic strategy is a general pattern discussed in the literature from a theoretical perspective (e.g. Andersen *et al.,* 2015; Ward and Follows, 2016; Chakraborty *et al.,* 2017): in general, small mixotrophs rely more in photosynthesis than larger species. Trophic optimization along the size spectrum include other mechanisms, which are difficult to grasp directly from the field data presented in this study. Yet, our analysis points to a size-dependent niche differentiation, as inferred by the formation of a bimodal size distribution. The above given numbers support an important role of this process within the trophic dynamics of plankton. The bimodality in the dinoflagellates size spectrum could according to theoretical considerations reflect an optimization of resource utilization. These ideas should be taken as hypothesis that may guide future studies such as on the trophic usage of the size spectrum by MTD.

Our niche differentiation scheme does not exclude other explanations for bimodality. Alternatively to the grazing pressure of large on small dinoflagellates, other heterotrophs, mainly copepods, could create the same disruptive selection that can be the origin of the dip around 30 μm ESD in dinoflagellate size spectra (Jansen *et al.,* 2006; Fileman *et al.,* 2010; Löder *et al.,* 2011). Other traits such as cell shape, motility and feeding type may also promote niche differentiation (Litchman *et al.,* 2015; Weithoff and Beisner, 2019). The schematic link between bimodality and mixotrophy proposed here should be further tested in future observational, laboratory and theoretical research.

## CONCLUSION

The increase in the mean size of mixotrophic dinoflagellates during the autumn bloom development is in line with the general species succession in temperate coastal seas. While size spectra of diatoms, coccolithophores, flagellates and unclassified plankton were unimodal, most size spectra of dinoflagellates were bimodal. Based on our size-based analysis of trophic linkages, we hypothesize that the regulation of mixotrophy by prey availability drives species succession of dinoflagellates, which induces bimodality in a niche differentiation process. The formation of a hidden food web including detritivory over the bloom development facilitates the direct transfer of nutrients and energy within the plankton community. This result may guide future research on the role of mixotrophy as a flexible mediation of diverse environmental stressors. For example, neither are physiological costs of mixotrophs quantified nor are trade-offs with other traits such as motility. This information would be necessary for models that seek to predict the onset and termination of dinoflagellate blooms.

## Supplementary Material

SM_fbac013
