# Supplementary material for: Phytoplankton and particle size spectra indicate intense mixotrophic dinoflagellates grazing from summer to winter
Source: J Plankton Res. 2022 Mar 14;44(2):224–40. doi: 10.1093/plankt/fbac013 (PMC8962713; doi:10.1093/plankt/fbac013)
Supplement: SM_fbac013 [file sm_fbac013.pdf]

Supplementary Material to Phytoplankton and particle size-spectra indicate intense mixotrophic dinoflagellates grazing from summer to winter

O. García-Oliva[1]*, F.M. Hantzsche[1,2], M. Boersma[2,3], and K.W. Wirtz[1]

[1] Helmholtz-Zentrµm Hereon, Max-Planck-Straße 1, 21502 Geesthacht, Germany.

[2] Alfred-Wegener-Institute Helmholtz-Zentrµm für Polar- und Meeresforschung, Biologischen Anstalt Helgoland, 27483 Helgoland, Germany.

[3] FB2, University of Bremen, Leobener-Straße, 28359 Bremen, Germany

* ovidio.garcia@hereon.de

SM1. Additional figures

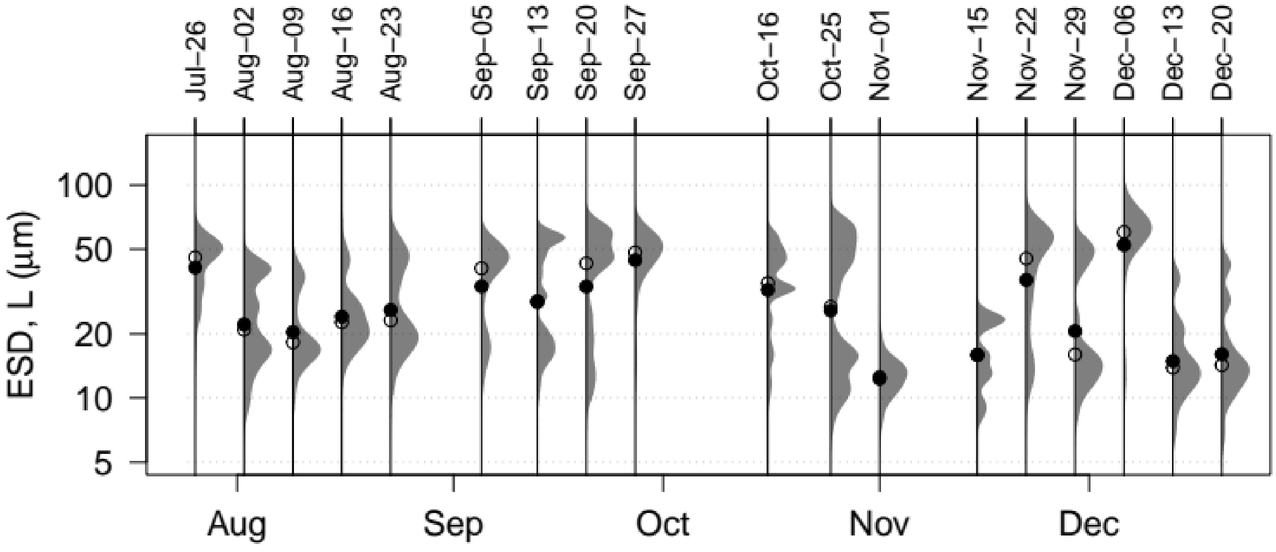

Figure SM-1: Reconstructed normalized biomass size-spectra of mixotrophic dinoflagellates (MTD) from Jul-26 to Dec-20. Solid black dots mark the mean size and empty black dots the median size for MTD.

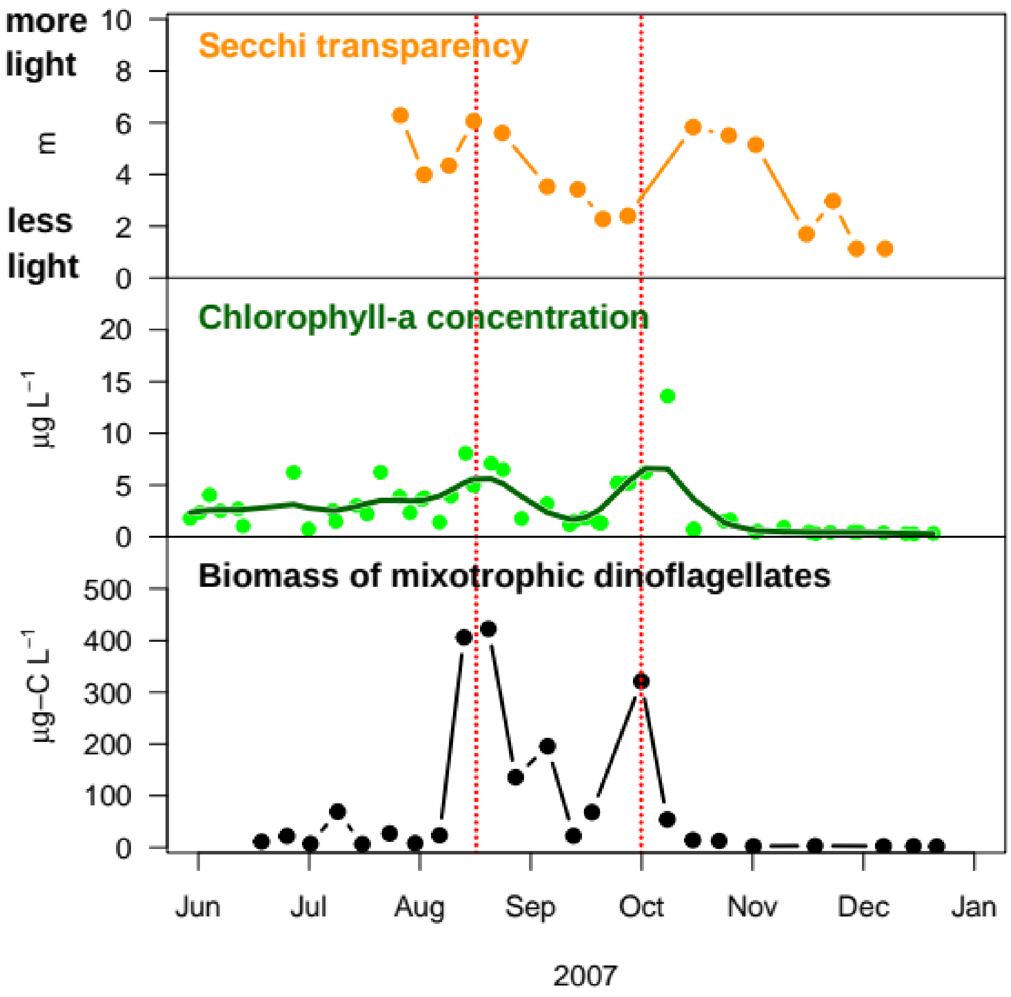

Figure SM-2: Biomass (black) and chlorophyll (green) of mixotrophic dinoflagellates after Hantzsche (2010) and Löder et al. (2012). The mean values from Jun to Dec were 80.1 µg-C L^{-1} and 2.8 µg L^{-1} for MTD biomass and chlorophyll-a, respectively. Secchi depth (orange) after Hantzsche (2010).

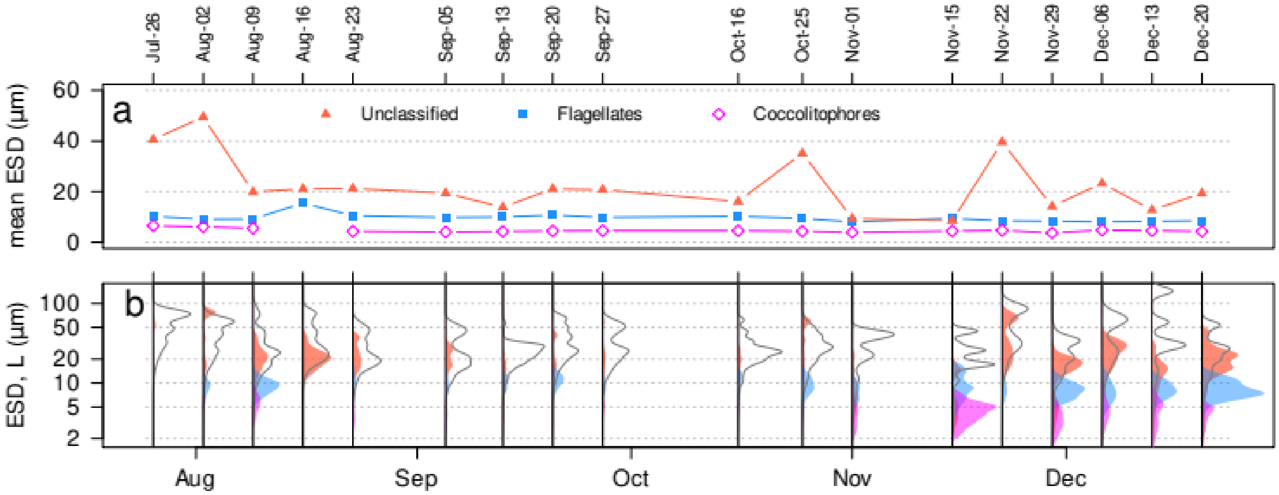

Figure SM-3: Flagellates, coccolitophores, and undefined phytoplankton dynamics from Jul-26 to Dec-20. (a) Mean cell equivalent spherical diameter (ESD) for unclassified plankton (red triangles), flagellates (blue squares), and coccolitophores (magenta diamonds). (b) Relative biomass-size spectrum of phytoplankton highlighting unclassified plankton (red), flagellates (blue) and coccolitophores (magenta). The spectra are scaled for better visibility: unclassified ×5, flagellates ×20, and coccolitophores ×50 relatively to the total phytoplankton spectrum (gray line).

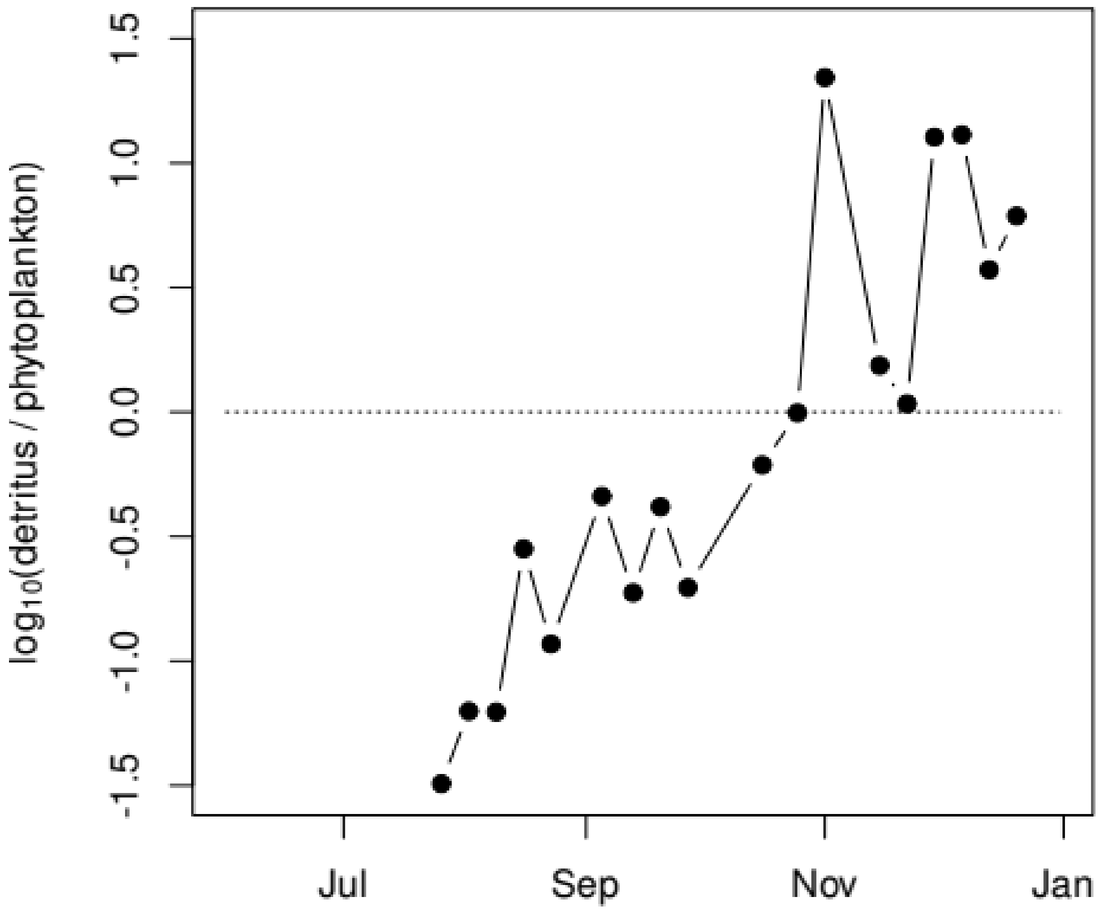

Figure SM-4: Detritus mass to phytoplankton biomass ratio within the feeding kernel of *A. sanguinea*.

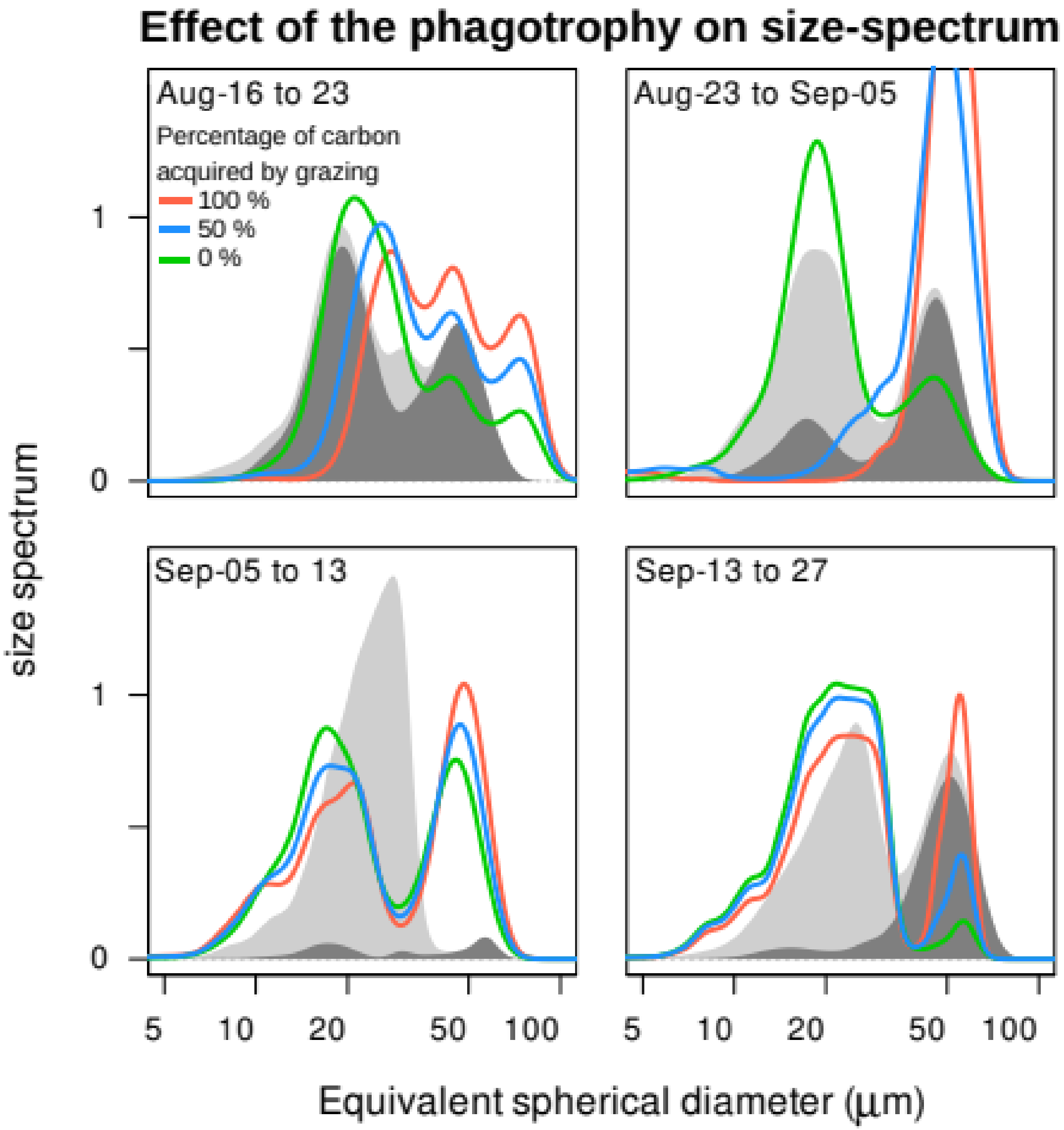

Figure SM-5: Biomass size spectra under three levels of carbon acquired by phagotrophy for mixotrophic dinoflagellates, which can be regarded as a proxy for the dependence of grazing on the total growth rate: red-100% phagotrophy (heterotrophy), blue-50% phagotrophy (balanced mixotrophy), and green-0% phagotrophy (autotrophy). The light and dark gray areas mark the observed normalized plankton biomass and normalized dinoflagellates biomass, respectively (García-Oliva et al. in preparation).

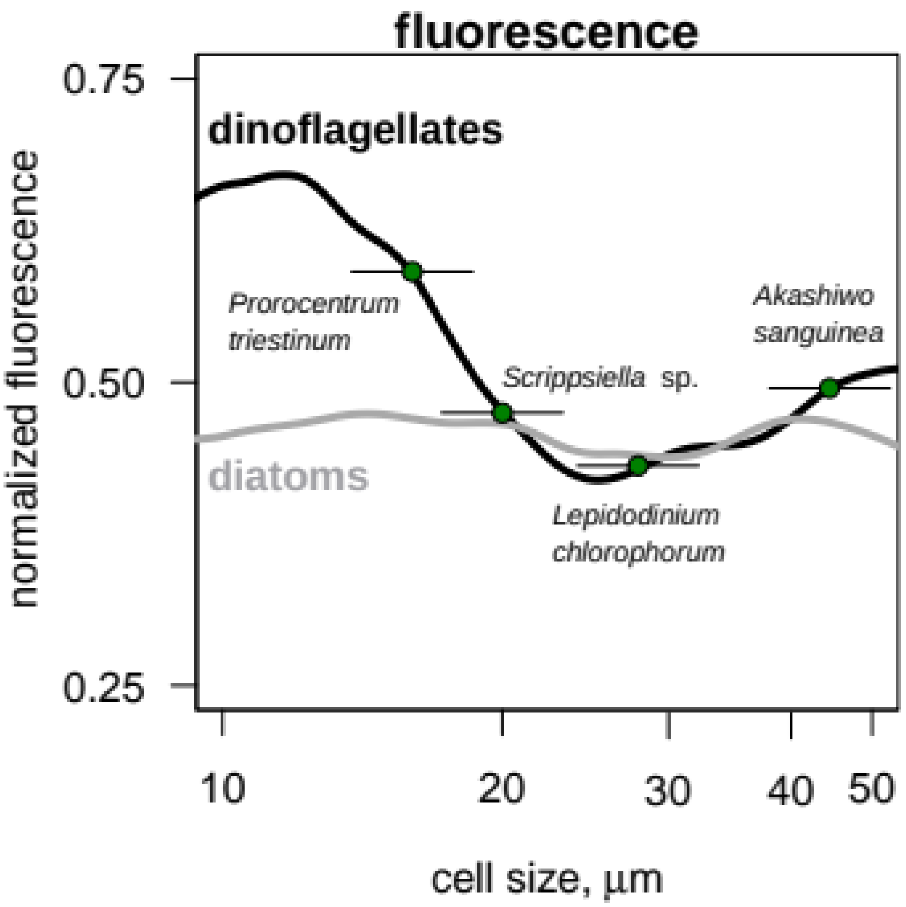

Figure SM-6: Normalized fluorescence peak (fluorescence signal triggered by the FlowCAM system over the maximum observed fluorescence peak value) depending on cell size for dinoflagellates (black line). Green dots mark the size of the four dominant species of dinoflagellates with the associated uncertainty value –approx. 0.15 log(µm)–. For comparison the diatom peak fluorescence as function of size is shown (gray line).

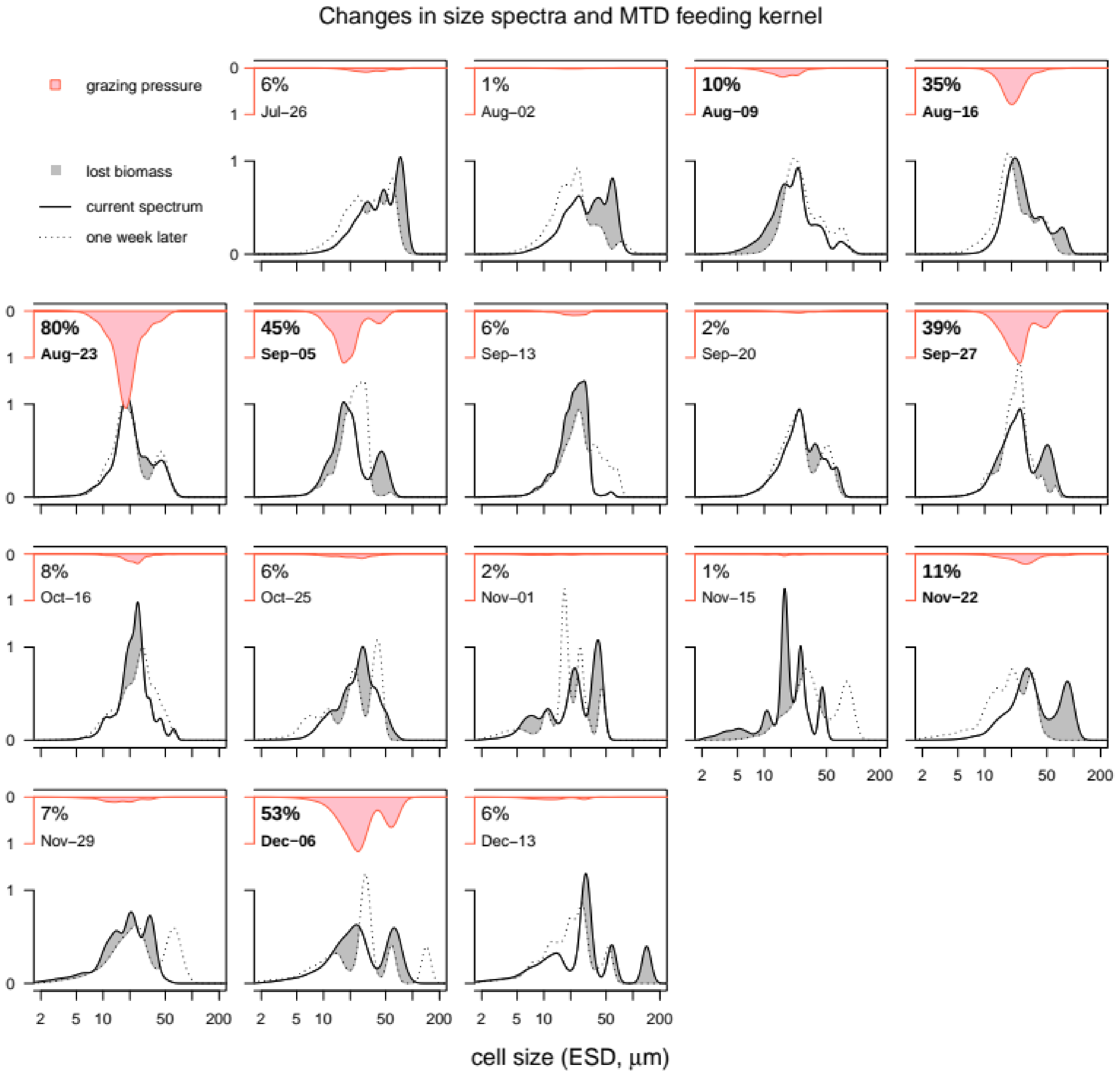

Figure SM-7: Changes in size spectra and potential grazing pressure of MTD. In boldface the dates when the dinoflagellates community was more than the 10% of the total biomass (value shown in the top left corner). The same information with no bias correction is shown in Fig. 3a.

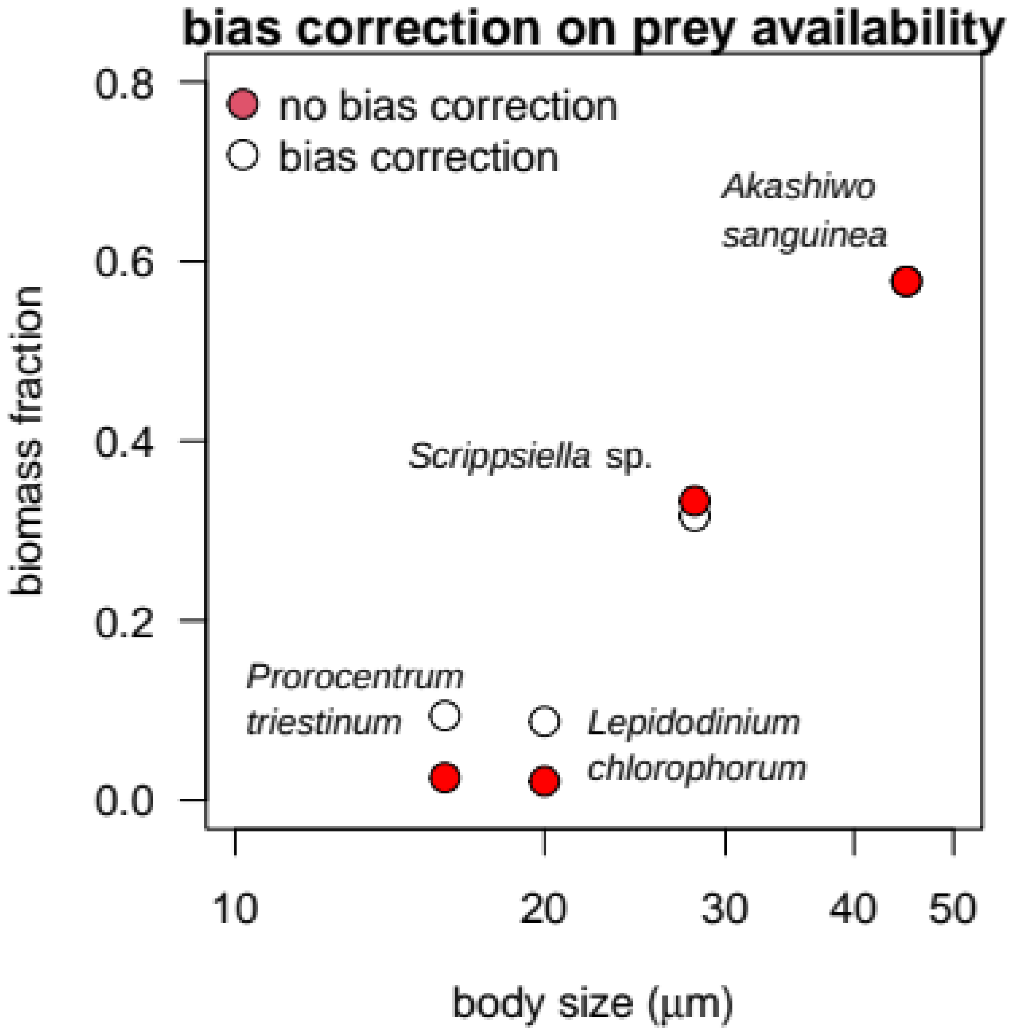

Figure SM-8: Comparison of mean prey availability (as calculated in Fig. 4b of the main text) with (void symbols) and without (solid symbols) bias correction.

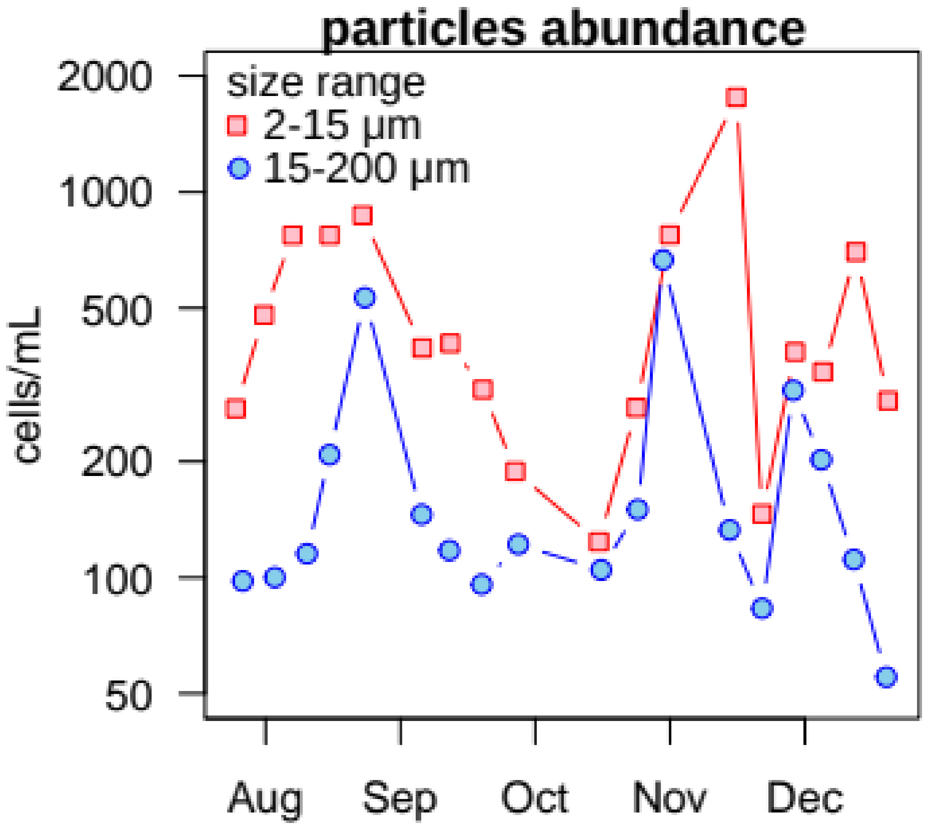

Figure SM-9: Particles abundance for the study period. (y-axis in log-scale.)

SM2. Validation and limitations of FlowCAM methodology

The acquisition of biomass size spectra is currently impaired by several limitations (Lombard et al. 2019). In our case, the FlowCAM system better captures larger rather than smaller particles (Sieracki et al. 1998). As mentioned in the Material and Methods section, we address this difficulty using two combinations of flowcell+magnification: (1) 100 µm + 20X for small particles (range 2-100 µm) and (2) 300 µm + 10X for larger particles (range 15-300 µm,). Further details on the methodology can be found in Hantzsche (2010). From the first combination, separated particle counts were kept for the ranges 2-15 µm and 15-100 µm. From the second combination, particles larger than 200 µm were removed from the dataset due the inverse filtration step conducted using a filter with 250 µm mesh size.

During our study period, the abundance of fluorescent particles in the range 2 to 15 µm were never below 100 cell/mL, reaching a maximum of approx. 2000 cell/mL before and during the dinoflagellate bloom. In comparison, the abundance of particles in the range 15-300 µm was approx. 100 cell/mL, with a maximum of 700 cell/mL during the dinoflagellate bloom (Fig. SM-9). Despite of the higher abundance of small particles, the chlorophyll concentration was not correlated neither with the particles in the 2-15 µm range nor with the one in the 15-300 µm range. However, particles >50 µm were significantly correlated with chlorophyll concentration ($r^2 = 0.67$, $p < 0.001$). From these comparisons we conclude that the biomass share of small cells (<50 µm) is not significant compared to the share of larger cells (>50 µm), at least for chlorophyll containing cells.

As a validation of our methodology, we compared the FlowCAM counts of fluorescent particles with microscopic counts for each size range. The FlowCAM counts of fluorescent particles underestimated the microscopic counts in a factor of approx. 10 for particles in the range 2-15 µm and in approx. 2.5 in the range 15-100 µm for the first flowcell+magnification combination, and in a factor of 2 for the range 15-300 µm for the second combination. We also observed significant correlations between FlowCAM and microscopic counts of flagellates

($r^2 = 0.25$, $p < 0.001$) and *Akashiwo sanguinea* ($r^2 = 0.64$, $p < 0.001$). These features were similarly reported for other FlowCAM applications (e.g. Hrycik et al. 2019) and can be summarized as follows: (1) despite the effort of using two different combinations of flowcell+magnification our methodology still presents a sampling bias toward larger cells, and (2) the FlowCAM counts are proportional to the microscopic counts, hence valid to assess the size structure of the phytoplankton community.

We applied two correction factors to the biomass spectra to ameliorate the effects of the sampling bias: 10 for the 2-15 μm fraction and 2 for the 15-300 μm fraction. However, due the small biomass per individual cell in the 2-15 μm fraction these corrections had little effect in our results, which can be noticed in the biomass size-spectra during the entire study period (Fig. SM-7): the bimodality of distributions, the temporal changes in size spectra, as well as the regions of maximum biomass loss are preserved after the bias correction. Bias correction did thus not affect our interpretations.

The little effect of the underestimation of small particles is also observed in the linear regression of the feeding loss index (FLI) as function of the dinoflagellates biomass share (x). The correlation of the feeding loss index with of dinoflagellates biomass share using the sampling bias correction is FLI = 0.47 x while for the uncorrected version is FLI = 0.49 x, both with similar levels of significance ($p < 0.001$) and the same standard deviation (0.03): the difference of the slopes is not significant (anova test, $p>0.1$). This result confirms our assumption of a minor influence of the 2-15 μm fraction on trophic dynamics.

The limitations of our method may lead to an underestimation of the phagotrophic activity of small MTD. Small dinoflagellates (*P. triestinμm* and *L. chlorophorum*) may in principle graze on small phytoplankton –i.e. flagellates–, especially before and during the onset of the dinoflagellates bloom. However, the low biomass share of flagellates and, in general, the low prey availability for small MTD (Fig. 4b in the main text) possibly renders phagotrophy a highly variable and scarce resource acquisition mechanism. The calculation of prey availability after bias correction does not substantially changed the previously

found values (Fig. SM-8). We conclude that the low prey availability for small MTD is a consequence of the biomass size distribution and not an artifact derived by our methodology. Furthermore, we presented the data with no correction factor to enhance the clarity of the presentation of our findings.

References to SM

Hantzsche, F.M. (2010) *An evaluation of the effect of food quality on heterotrophic protists with a critical assessment of a new measuring technique (FlowCAM)*. PhD thesis, Christian-Albrechts-Universität Kiel.

Hrycik, A.R., Shambaugh, A. and Stockwell, J.D. (2019) Comparison of FlowCAM and microscope biovolume measurements for a diverse freshwater phytoplankton community. *J. Plankton Res.*, **41**, 849–864.

Lombard, F., Boss, E., Waite, A.M., Uitz, J., Stemmann, L., Sosik, H.M., Schulz, J., Romagnan, J.B., Picheral, M., Pearlman, J., Ohman, M.D., Niehoff, B., Möller, K.O., Miloslavich, P., Lara-Lopez, A., Kudela, R.M., Lopes, R.M., Karp-Boss, L., Kiko, R., Jaffe, J.S., Iversen, M.H., Irisson, J.O., Hauss, H., Guidi, L., Gorsky, G., Giering, S.L.C., Gaube, P., Gallager, S., Dubelaar, G., Cowen, R.K., Carlotti, F., Briseño-Avena, C., Berline, L., Benoit-Bird, K.J., Bax, N.J., Batten, S.D., Ayata, S.D. and Appeltans, W. (2019) Globally consistent quantitative observations of planktonic ecosystems. *Front. Mar. Sci.*, **6**, 196.

Sieracki, C.K., Sieracki, M.E. and Yentsch, C.S. (1998) An imaging-in-flow system for automated analysis of marine microplankton. *Mar. Ecol. Prog. Ser.*, **168**, 285–296.
